# Study of Polyphenol Content and Antioxidant Properties of Various Mix of Chocolate Milk Masses with Different Protein Content

**DOI:** 10.3390/antiox9040299

**Published:** 2020-04-03

**Authors:** Bogumiła Urbańska, Tomasz Szafrański, Hanna Kowalska, Jolanta Kowalska

**Affiliations:** 1 Institute of Food Sciences, Department of Technology and Food Evaluation, Warsaw University of Life Sciences, 159c Nowoursynowska St., 02-776 Warsaw, Poland; szafranski.tomasz@hotmail.com (T.S.); jolanta_kowalska@sggw.pl (J.K.); 2 Institute of Food Sciences, Department of Food Engineering and Process Management, Warsaw University of Life Sciences, 159c Nowoursynowska St., 02-776 Warsaw, Poland; hanna_kowalska@sggw.pl

**Keywords:** conching, milk chocolate, milk powder, protein, polyphenols, antioxidant activity

## Abstract

The aim of the study was to analyze the antioxidant character of conched chocolate milk masses, taking into account different protein content in milk. For the study, cocoa liquor obtained from roasted and unroasted cocoa beans from different regions, as well as milk powder obtained by spray and cylindrical drying were used. The analysis that was carried out showed that the protein content of powdered milk products ranged from about 11.6% (*w/w*) to over 31% (*w/w*). Lower content of polyphenols and lower antioxidant activity were shown in the masses to which the addition of milk with higher protein content was applied. The analysis of antioxidant character of chocolate milk masses showed higher total polyphenols content in masses prepared from unroasted cocoa beans liquor.

## 1. Introduction

Cocoa beans are seeds of the tropical Theobroma cacao tree. There are three best known varieties of this plant: Forastero, which covers about 95% of the world’s cocoa production and is most commonly used to produce chocolate; Criollo, which is the most exclusive but at the same time the least cultivated variety due to its susceptibility to diseases; and Trinitario, a hybrid combining the characteristics of Criollo and Forastero, with an intense chocolate aroma, with a hint of wine, more resistant to diseases and pests [1,2].

Many studies and numerous publications confirm that cocoa beans are a raw material carrying a powerful load of antioxidants, valued in today’s diets mainly for their antiatherogenic, antiradical, and anticancer properties [3,4,5,6]. The genotype of cocoa beans, the region in which they are grown, the environmental conditions, as well as the conditions and parameters of applied technological operations, especially fermentation, drying, roasting, and conching, have a significant influence on the formation of sensory characteristics and antioxidant properties of cocoa bean processing products [7,8,9,10]. As numerous studies have shown, the most degrading content of phenolic compounds is the roasting stage—high-temperature heating of beans or cocoa shots, which significantly affects the antioxidant potential of chocolates [11,12,13,14,15]. The use of less processed cocoa beans (omitting the roasting process) for the production of chocolate mass may result in a product with a higher content of polyphenols and higher antiradical activity, as well as positively change the nutritional and health value. Therefore, interest in cocoa mass obtained from unroasted cocoa beans [2] has increased in recent years.

Conching is one of the main and most important stages in the chocolate production process. It consists of stirring and aerating the chocolate mass with simultaneous heating at a certain temperature (>40 °C) [16,17]. Conching plays an important role in the development of taste by removing undesirable volatile compounds and moisture and by obtaining a homogeneous mass of appropriate particle size [9]. The time of conching, which is unfavorable from the point of view of production efficiency, is significantly influenced by the temperature and speed of mixing [18]. These parameters are also important for the course and intensification of the Maillard reaction as well as the Strecker degradation reaction [19]. The choice of process parameters is adapted to the type of product, its composition, and the production capacity of the plant. In order to limit or prevent the Maillard reaction it is recommended to carry out the process of conching milk masses at a temperature not exceeding 50 °C [9]. The Maillard reaction is affected by many aspects, including temperature, pH, water content, duration of heating, type of reactant, oxygen, ratio of amino acid to sugar, metals, and reaction inhibitors [20].

Few studies indicate that the percentage of the conching process has no significant effect on the content and phenolic system, as well as on the antioxidant activity [1,19,21,22]. The results obtained by Di Mattia, et al. [23] even indicate an increase in the antioxidant activity of conched masses, mainly due to the growing potential of melanosides.

The legal act establishing guidelines and minimum requirements for a product that can be called chocolate is Directive 2000/36/EC of the European Parliament and of the Council of 23 June 2000 [24]. It indicates that chocolate is a product obtained from cocoa products and sugar, containing not less than 35% (*w/w*) total dry cocoa solids and a minimum of 18% (*w/w*) cocoa butter and 14% (*w/w*) dry non-fat cocoa solids. The original chocolate recipe is based on only three ingredients: cocoa mass, cocoa butter (fat), and sugar. However, with the development of the chocolate market, other raw materials and additives such as emulsifiers, which stabilize the structure and consistency of chocolate and flavors that enhance its taste and smell, started to be used [3,25]. An example of another raw material used in the production of chocolate is powdered milk, which was first used in 1875 and gave the chocolate a more velvety texture and a pleasant sweet and milky character. Two types of milk powder are used to produce chocolate: roll-dried and spray-dried. Roll-cured milk is more preferred because the chocolate mass produced from it ensures optimum viscosity, while spray-dried milk-based chocolate mass has a much higher viscosity. This is due, among other things, to physical differences in the material. Powder dried on a cylinder has a high content of free fat (at the level of about 90%), while spray-dried powder has below 10%. Powder dried on rolls usually has a larger average particle size of about 150 µm and a small vacuole volume, while spray-dried powder has a smaller particle size—about 70 µm—and a high vacuole volume [26].

The protein content in bitter chocolate is at the level of 5–15% (*w/w*) and it is the protein derived from cocoa beans. Peptides are currently considered important bioactive constituents of food but the potential biological activities of oligopeptides found in cocoa are not sufficiently researched in cocoa literature. This protein is characterized primarily by a low degree of amino acid release during digestion, which results in low nutritional importance of this component in our diet. In the case of milk chocolates, however, the protein content is primarily determined by the amount of milk powder added, in which the content of this component oscillates between 25% and 30% (*w/w*). Milk protein is characterized by much better assimilability compared to cocoa, which translates into increased nutritional importance of this protein in the diet of the consumer [27,28]. According to Maidannyk, et al. [29], milk powder proteins are susceptible to caking and adhesion and may be highly insoluble. Therefore, storage conditions of the powder are an important factor responsible for such problems.

Most studies analyze the antioxidant properties of bitter chocolates, which are a rich source of these compounds. Due to lower content of cocoa mass and fat-free components of cocoa bean processing, milk chocolates are characterized by much lower antioxidant potential [30]. Moreover, most of the studies concern chocolates obtained in the traditional technological cycle and include mainly the influence of the origin of raw materials and the roasting process on the physical and chemical properties of these products. The results of these studies are inconclusive and although they tend to confirm the positive correlation between polyphenol content and antioxidant activity, as well as the degrading effect of roasting on polyphenol content, there are studies that do not confirm these results [22]. As shown by the results of the work involving the evaluation of consumer preference for chocolate, consumers are most likely to reach for milk chocolates.

However, research on milk chocolates is not conducted on a large scale, especially in terms of evaluating their antioxidant potential. They are mainly produced using milk obtained from spray drying and such products are the subject of most studies and publications. Moreover, chocolates called “raw”—produced without roasting—have appeared on the market. There is little research into these products. As well as few studies on the effect of conching on antioxidant properties have been published. Therefore, it seems reasonable to attempt to assess the effect of conching on the antioxidant potential of chocolate milk masses, taking into account the protein content of milk powder obtained by spray or cylindrical drying, as well as to analyze the subject of research after using roasted and unroasted cocoa pulp.

The aim of the study was to investigate the relationship between the protein content of milk powder and cocoa mass and the antioxidant potential of chocolate milk masses after conching.

## 2. Materials and Methods

### 2.1. Test Material

The research material was 19 milk powders differing in production technologies (Table 1) and the raw materials used to prepare chocolate milk masses—cocoa liquors from 3 manufacturers, differing the country of origin and in the process of processing the beans from which they were made and selected powdered milk (Table 2).

Milk powders and cocoa masses were obtained from the chocolate products manufacturers.

The first stage of the research was to carry out the analysis of dry matter and protein content in powdered milk according to the research methodology described in the subchapter of analytical methods 3.1 and 3.2, respectively.

On the basis of the results obtained, seven milk products were selected for further studies—3, 4, 5, 13, 14, 15, and 18 differing in protein and dry matter content. The selected milk was marked with new codes respectively: 3—Ml_1, 4—Ml_2, 5—Ml_3, 13—Ml_4, 14—Ml_5, 15—Ml_6, and 18—Ml_7 (Table 2).

The samples marked with the codes Ml_1, Ml_2, Ml_3, Ml_6, and Ml_7 were spray-dried whole milk powders, differing in protein content (Table 1). On the other hand, the milk Ml_4 and Ml_5 differed from the other compositions (they were mixtures of milk with sugar) and were produced using the cylindrical method.

### 2.2. Technological Process

Chocolate milk masses were prepared by mixing (Table 3):

- Cocoa masses—16.2%,
- Cocoa butter—12.3%,
- Sugar—50%,
- Milk powder—18.0%,
- Whey—3.2%,
- Lecithins—0.3%.

Chocolate milk masses were prepared from ingredients weighed and mixed at controlled temperature and time conditions in the Termomix device (Vorweck, Germany)—the household appliances. The first step was to liquefy a weighed portion of cocoa butter at 45 °C. Then the fat was poured into another container. The cocoa liquor was liquefied at 55 °C. The sugar, milk powder, whey, and 10% cocoa butter were added to the liquefied cocoa liquor. The ingredients were mixed for 10 min at a constant temperature of 45 °C, then residual cocoa fat and lecithin were added. The final stage was the proper conching, i.e., slow mixing of the mass at 55 °C for 1 h.

## 3. Analytical Methods

The reagents used for the analyses were purchased from Sigma Aldrich Company.

### 3.1. Determination of Dry Matter Content

The dry matter content was determined in all samples of milk powder and cocoa liquor. This analysis consisted in the evaporation of water from the material tested during the drying process, followed by weight determination of the residue (dry matter).

The weight of test material was dried to constant mass at 105 °C for 3 h in the laboratory chamber oven type SUP-65 WG manufacturer WAMED. Samples of cocoa liquor and chocolate masses were dried with sand to increase the evaporation surface. The dry matter content was calculated from the difference of sample weight before and after drying. The arithmetic mean of three parallel repetitions for each sample was taken as the final result.

### 3.2. Determination of Protein Content

The determination of protein content was performed in all samples of milk powder and cocoa liquor.

The protein content was determined by the Kjeldahl method. The principle of the method consisted in mineralization of samples with concentrated sulphuric acid (VI) in the presence of a catalyst (selenium-copper mixture) in the Buchi 426 Dugestion Unit. Under these conditions, the protein nitrogen was converted to an ammonium ion, which after alkalization was distilled in the form of ammonia and bound in excess boric acid in Buchi’s distillation unit B-316. The ammonia solution was determined by potentiometric titration with 0.1 N hydrochloric acid standard solution. The nitrogen content in the sample was calculated on the basis of the volume of 0.1 N of hydrochloric acid standard solution used for titration, knowing that 1 cm^3^ of 0.1 N of hydrochloric acid solution corresponds to 0.0014 g of nitrogen. Nitrogen was converted into protein using a multiplier calculated from the average nitrogen content in proteins, which is 16% (100:16 = 6.25). The determination was carried out in three consecutive repetitions.

### 3.3. Determination of Total Polyphenols Content by the Folin–Ciocalteu Method

Total polyphenol content was determined by Folin–Ciocalteu’s method [31,32]. Based on preliminary tests, a 70% acetone solution was used as a solvent to prepare extracts. The extracts were prepared by weighing about 5 g of crushed test material into 300 mL grinding conical flasks and adding 100 mL of 70% acetone (*v/v*). The samples were then shaken for 30 min in a Multi-Shaker PSU 20 Biosan shaker. Following this procedure, the solutions were filtered through the corrugated filters into 100 mL grinding flasks. In order to determine the total polyphenol content, 300 µL of the extract was taken from the tubes, and 4.15 mL of deionized water, 500 µL of 20% sodium carbonate solution, and 50 µL of the Folin–Ciocalteu reagent were added. The blank sample was prepared by sampling: 300 µL of the extraction solution, 4.15 mL of deionized water, 500 µL of the 20% sodium carbonate solution, and 50 µL of the Folin–Ciocalteu reagent. Absorbance was measured at 700 nm on a Shimadzu UV-160A spectrophotometer. The apparatus was zeroed to a blank. In order to calculate the total polyphenol content, a standard curve was prepared. The standard curve was plotted for chlorogenic acid for various concentrations (0, 25, 50, 75, and 100 µL) used in absorbance measurements. Based on the results obtained, the graphical dependence of the absorbance of the solution on the amount of gallic acid contained in it was plotted. The total polyphenol content was calculated on the basis of the calibration curve and expressed in gallic acid equivalent in mg per 100 g d.m. (mg GAE∙100 g^−1^ of the product Two extracts from each mass were made and polyphenols content was determined in three parallel repetitions for each extract. The average of six repetitions for each mass was considered the final result.

### 3.4. Determination the Ability of Extracts to Inactivate Stable DPPH Radicals

The extracts were prepared by weighing 5 g test material to conical flasks (300 mL) and adding 100 mL of 70% acetone (*v/v*). The samples were then shaken for 30 min in a Multi-Shaker PSU 20 Biosan shaker. Following that, the solutions were filtered through the corrugated filters into 100 mL flasks. Acetone extract (1 mL), acetone solution (3 mL), and added DPPH solution (1 mL) were taken to determine the appropriate sample. Acetone solution (4 mL) and DPPH solution (1 mL) were collected for the control sample. The samples were mixed and left to stand for 30 min, then absorbance was measured on the NOVASPEC II Pharmacia spectrophotometer (apparatus were zeroed for the blank test) at a wavelength of 517 nm in glass cuvettes with a diameter of 1 cm [33,34].

The antioxidant activity of the extracts against DPPH was calculated using the formula:

Act. = [(Ak − As)/Ak] × 100% (1)

where Act.—antioxidant activity (%); Ak—the absorbance of the control sample; and As—absorption of the specific sample.

### 3.5. The Statistical Analysis

The statistical analysis of results was performed in the Statistica 13.0. Program by using one- and two-factor analysis of variance at significance level α = 0.05 to determine differences between the content of polyphenolic compounds and the antioxidant ability the test samples of chocolate milk masses, taking into account the effect of the protein content of milk powder. Significant differences between means were determined through Tukey’s tests. The correlation matrix was analyzed to determine the relationship between polyphenol content and antioxidant activity.

## 4. Results and Discussion

### 4.1. Selection of Raw Materials for Chocolate Milk Masses Production

The selection of components differentiating the mixtures was carried out on the basis of their own characteristics and the results of analyses to which they were subjected (Table 1 and Table 4).

For preparation of milk masses three cocoa liquor were used: Ch_1, Ch_2, and Ch_3 (Table 4) and seven of tested powdered milk: Ml_1, Ml_2, Ml_3, Ml_4, Ml_5, Ml_6, and Ml_7 (Table 1 and Table 2). The remaining part of the composition consisted of ingredients such as cocoa fat, sugar, whey, and lecithin.

The protein content in the tested cocoa masses oscillated around 13.6–14.6% (*w/w*). The values obtained in this study are also presented by the studies of Jumnongpon, et al. [35] and Torres-Moreno, et al. [36], who determined the protein content in cocoa beans from which cocoa mass is produced at the level of 13–20% (*w/w*). Selected pulp differed in producer, origin, and type of grain from which they were prepared, while in the case of dry substance and protein content they had similar parameters (Table 4).

Proteins are important bioactive food ingredients, but the potential biological activity of oligopeptides found in cocoa is insufficiently researched. Biologically active or functional proteins come primarily from food and have, in addition to their nutritional value, a physiological effect on the body [37].

In cocoa, peptides are formed naturally during the fermentation of cocoa beans and are considered important flavor precursors. The peptides come from two main protein fractions—globules, consisting of a storage protein resembling vicilin and albumin, which exhibit trypsin inhibitory properties. Cocoa proteins during natural cocoa fermentation are split into hydrophilic and hydrophobic peptides as well as amino acids by autolysis of two endogenous enzymes: aspartic endoprotease and carboxypeptidase activated by microbial metabolites such as acetic acid [38]. Milk proteins include complex groups of proteins: caseins (αs1-, αs2-, β-, and κ- casein), whey proteins (β-lactoglobulin, α-lacto-albumin, albumin serum, and immunoglobulin), and protein envelopes of fat globules. Structural differences within milk protein molecules affect their properties [39]. It is assumed that β-casein is the most effective stabilizer among milk proteins as it reduces the surface tension most [40].

According to Christian Vásquez, et al. [41] milk proteins can act as surfactants and stabilizers that are able to induce the formation of spherical micellar aggregates in the chocolate structure. Moreover, spray drying technology can induce changes in proteins that affect hydrophobicity, solubility, and denaturation [42,43,44]. Milk selected for preparation of milk chocolate masses contained from 11.61 to 31.1 mg of protein in 100 g of powder (Table 1). The selection was made in such a way that protein content in the analyzed milk was differentiated. This allowed for comparative analysis and evaluation of the dependence between phenol potential and protein content.

Antioxidants prevent formation of free radicals or support their removal. The milk contains several antioxidant factors such as vitamins and enzymes. Possible antioxidant activity of milk proteins and their hydrolysates has also been shown. It has been reported that peptides produced from milk protein digestion have an antioxidant effect. According to Pihlanto [45], antioxidant peptides derived from milk consist of 5–11 amino acids, including hydrophobic amino acids, proline, histidine, tyrosine, or tryptophan in the sequence.

The cocoa liquor and milk used in the study were subjected to a technological process to obtain 21 chocolate milk masses, which were tested for polyphenols content and antioxidant activity against free radicals of DPPH.

### 4.2. Results of Polyphenols Content Determination in Chocolate Milk Masses

The presence of polyphenols in milk depends on the animal’s diet and may affect the preservative effect of milk components [46].

**Figure 1:**
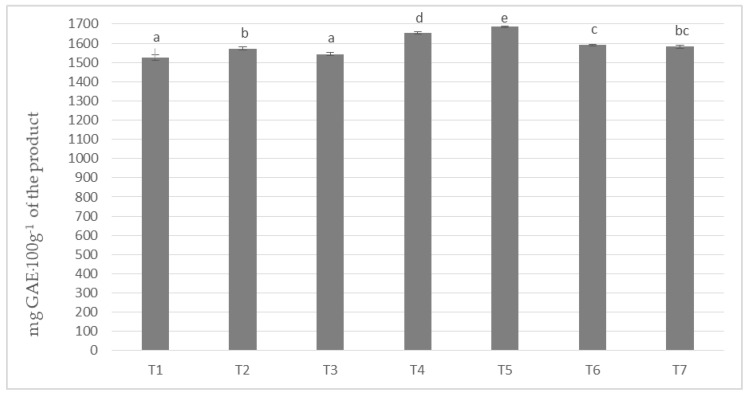
Polyphenols content in chocolate milk masses prepared from cocoa liquor from Peru (the same letter means that there are no statistically significant differences between the analyzed products at a confidence level of α = 0.05; the abbreviations used in the graph are described in Table 3).

In milk masses made on the basis of cocoa liquor obtained from unroasted beans from Peru (Ch_1) and seven milk/milk powder mixes, the polyphenols content was obtained from about 1525 to about 1685 mg calculated as gallic acid in 100 g of product (Figure 1). The highest content of phenolic compounds was achieved by the T-5 sample with the addition of milk Ml_5 (protein content 13.19%) and the lowest by T1 based on whole milk powder Ml_1 (protein content 30.35%). The differences in the content of polyphenols were statistically significant, as indicated by five homogeneous groups.

**Figure 2:**
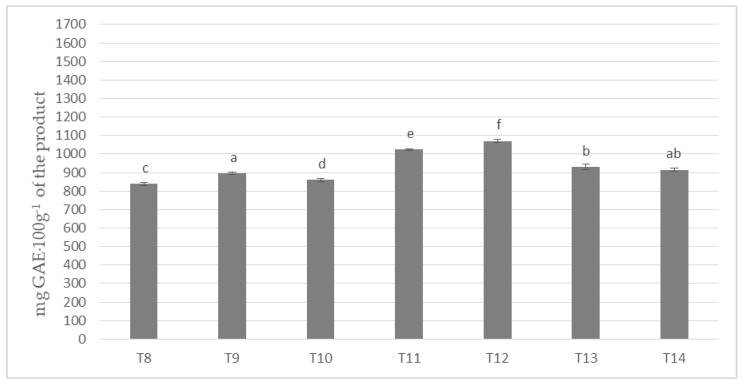
Polyphenols content in chocolate masses prepared from Ivory Coast cocoa liquor (the same letter means that there are no statistically significant differences between the analyzed products at a confidence level of α = 0.05; the abbreviations used in the graph are described in Table 3).

In the investigated milk masses prepared on the basis of cocoa liquor bean from the Ivory Coast (Ch_2) in combination with seven different milk powders the results of polyphenols content on the level from about 838 to almost 1071 mg per 100 g of product were obtained (Figure 2).

The highest content of polyphenols was determined for the mass of T-12, to which the milk mixture Ml_5 (dried by cylinders method) was used, whereas the lowest for the mass of T-8, to which spray-dried whole milk was applied (Ml_1). All masses formed six homogeneous groups, which prove their statistically significant differentiation in terms polyphenols content. Only T-14 masses did not differ significantly from T-9 and T-13 samples. In the presented values a specific order of increase in the content of phenolic compounds from T-8, through T-10, T-9, T-14, T-13, T-11, to T-12 chocolate was observed. It is worth emphasizing that T4 and T5, as well as T11 and T12 masses were characterized by the highest content of polyphenols, and Ml_4 (protein content 18.9%) and Ml_5 (protein content 13.19%) milk with the lowest protein content was used to produce them.

**Figure 3:**
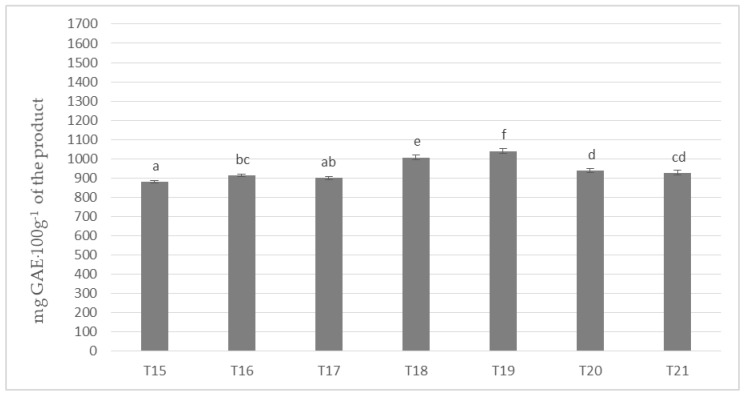
Polyphenols in chocolate milk masses prepared from Ghana cocoa liquor (the same letter means that there are no statistically significant differences between the analyzed products at a confidence level of α = 0.05; the abbreviations used in the graph are described in Table 3).

Figure 3 shows the results of the determination of polyphenols content in masses obtained with the use of cocoa liquor made from cocoa beans grown in Ghana (Ch_3). Invariably, the mass with the highest content of the determined compounds remained the one with the addition of milk Ml_5 (T5 and T12 on Figure 1 and Figure 2) and the one with the lowest one with the addition of whole milk powder Ml_1. A similar tendency to the polyphenol content was found as in the samples with the addition of cocoa liquor from the Ivory Coast. However, the differences between the masses were much less pronounced, but nevertheless formed six homogeneous groups. By comparing the results of the masses in Figure 3 with those produced from Ch_2 cocoa liquor (Ivory Coast), an average of 3–5% higher total polyphenol content could be observed in each sample in favor of those produced from beans from Ghana, regardless of the milk used. However, it is noteworthy that Ghanaian cocoa liquor beans were found to have a lower polyphenol content of approximately 6% compared to the values determined for Ivory Coast cocoa liquor bean. However, the protein content of the Ch_2 cocoa liquor was about 3% higher than that of the Ch_3 cocoa liquor (Table 4). The results obtained may confirm the literature data describing interactions between polyphenols and proteins present in the chocolate matrix. This is mainly due to the induction by proteins of the formation of spherical micellar aggregates, which close polyphenolic compounds limiting their availability.

Analyzing the results obtained for all 21 chocolate milk masses, it was noted that the amount of polyphenols contained in them oscillated between 35% and 50% of the values obtained for the cocoa liquor from which they were obtained. This is mainly due to the recipe in which cocoa mass constitutes less than 2/3 of the raw material composition of the finished chocolate milk mass. Additionally, not only the cocoa liquor (although certainly in the majority) brings an additional antioxidant character to the chocolate milk masses, because, as indicated by the studies of Ertan, et al. [47], whole milk powder may contain from about 50 to even 100 mg of polyphenols converted into gallic acid in 100 g of product. Summarizing these values in the finished product, which is a milk mass, it is possible to explain a polyphenol content of 1000–1500 mg in chocolates based on roasted cocoa liquor bean and correspondingly more in chocolates prepared from unroasted cocoa liquor bean. While the values for dairy products based on unroasted cocoa liquor bean were within the assumed range, this was not the case for roasted cocoa liquor bean. This could result in a small extent from the treatments (in the process of conching) to which the milk mass was subjected, losing between 5% and 10% of phenolic compounds as indicated by Di Mattia, et al. [1]. However, in the majority of cases, this loss was probably the result of possible interactions between polyphenols and proteins contained in milk and whey powder, intensified mixing time to which chocolate masses are subjected. As Gallo, et al. [48] points out, polyphenol–protein interactions may occur in milk chocolates, resulting in compounds that are inaccessible to the components of the reaction, while limiting their availability and in vitro oxidative potential. These studies showed a 15% decrease in the assay of polyphenols combined with casein, a 25% decrease in combination with whey proteins and almost 40% decrease in combination with pure ß-lactoglobulin (the main whey protein of milk). The results of these studies may explain the trends obtained in the studies carried out in this study, considering that the results obtained after statistical analysis confirmed a correlation of −0.66 between protein and polyphenol content in chocolate milk masses.

The comparative analysis of polyphenol content in all analyzed milk chocolate masses was 13 homogeneous groups, which indicates a large diversity of tested samples (Table 5). Considering that the protein content in chocolate pulp used to make the masses was at a similar level, and every third sample contained the addition of the same milk, such a large number of homogeneous groups indicate the complexity of factors shaping the antioxidant potential.

### 4.3. Results of Determination of Antioxidant Activity in Chocolate Milk Masses

Whey proteins may have an antioxidant activity, while heat treatment at very high temperature has no clear effect on the antioxidant potential of milk [49].

Antioxidant potential (AP) is an important nutritional property of food, as increased oxidative stress has an effect on most diet-related chronic diseases. In dairy products the protein fraction shows antioxidant activity, especially casein. Pihlanto study [45] reported that peptides produced from milk protein digestion have an antioxidant effect. Antioxidant peptides derived from milk consist of 5–11 amino acids, including hydrophobic amino acids, proline, histidine, tyrosine, and tryptophan in sequence.

The milk contains several antioxidants: naturally occurring vitamins (i.e., E and C), β-carotene, enzymatic systems, serum albumin, and lactoferrin, which act as chelating agents, iron-binding glycoprotein, and the activity of free radical scavenging by amino acids such as tyrosine and cysteine [45]. Therefore, polyphenols may be the main contributor to the antioxidant capacity of the food matrix [44].

The solubility of milk protein in water may be closely related to the oxidative status as oxidation improves interactions and protein aggregation [50], which reduces the solubility of milk powder [51].

**Figure 4:**
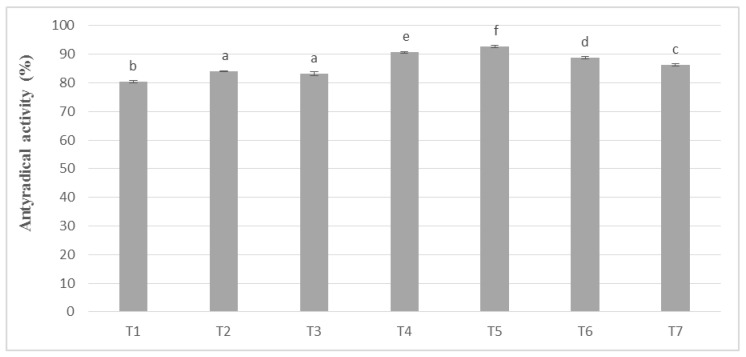
Antioxidant activity in chocolate milk masses prepared from unroasted cocoa liquor from Peru (the same letter means that there are no statistically significant differences between the analyzed products at a confidence level of α = 0.05; the abbreviations used in the graph are described in Table 3).

In these studies, the antioxidant activity remained in the same upward trend as polyphenols. The results of the phenolic potential expressed as antioxidant activity in the masses obtained from unroasted cocoa beans are shown in Figure 4. However, it was noticeable, as in the case of the determination of phenolic compounds, that higher values, on average by 8–10%, were obtained for the masses obtained from unroasted cocoa beans. The obtained values were subjected to statistical analysis, on the basis of which the masses were assigned to seven homogeneous groups. No significant differences were observed between T2 and T3 masses, prepared successively from whole milk powder Ml_2 and Ml_3, containing 27.44% and 29.14% (*w/w*) protein, respectively.

In chocolate milk masses prepared from unroasted cocoa liquor bean from Peru, the results of determination of antioxidant activity against free radicals of DPPH were 70–80% (Figure 4). The highest activity was characterized by T5 mass with the addition of milk mixture Ml_5, while the lowest T-8 mass with addition of whole milk powder Ml_1, which coincides with the dependence obtained during polyphenols determination. Similar observations were also observed in the significance of differences in the levels of antioxidant activity as, as well as in the case of polyphenols content. Chocolate milk masses from the Ivory Coast cocoa liquor bean formed six homogeneous groups from which no significant differences between the T14 and T9 and T13 mixture were found.

**Figure 5:**
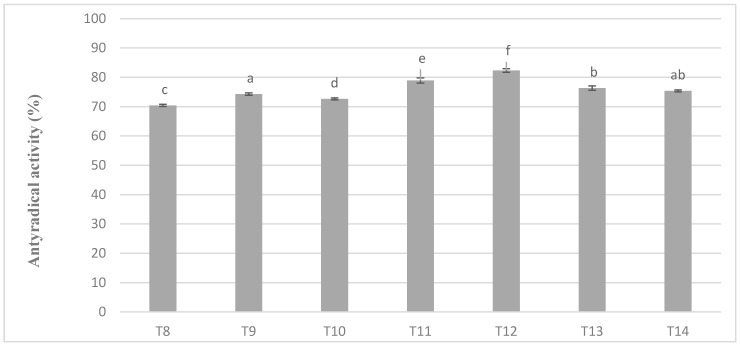
Antioxidant activity in chocolate milk masses prepared from Ivory Coast cocoa liquor (the same letter means that there are no statistically significant differences between the analyzed products at a confidence level of α = 0.05; the abbreviations used in the graph are described in Table 3).

In chocolate milk masses prepared from roasted cocoa liquor bean from Ivory Coast the results of determination of antioxidant activity against free radicals of DPPH were 70–80% (Figure 5). The highest activity was characterized by T12 mass with addition of milk mixture Ml_5, while the lowest T-8 mass with addition of whole milk powder Ml_1, which coincides with the dependence obtained during polyphenols determination. Similar observations were also observed in the significance of differences in the levels of antioxidant activity as well as in the case of polyphenols content. Milk masses from the Ivory Coast cocoa liquor bean formed six homogeneous groups from which no significant differences between T14 and T9 and T13 mixture resulted (Figure 5).

**Figure 6:**
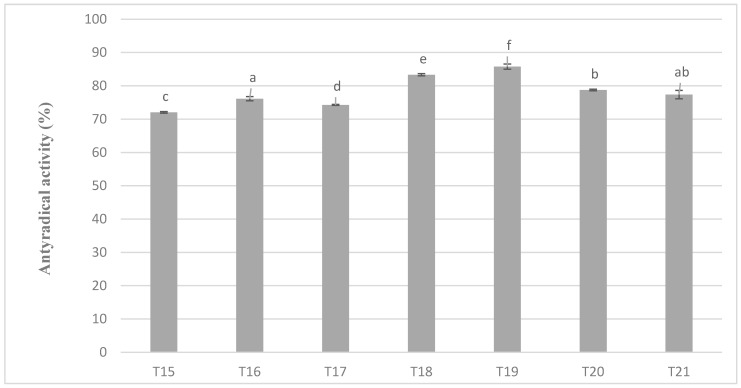
Antioxidant activity in chocolate milk masses prepared from cocoa liquor from Ghana (the same letter means that there are no statistically significant differences between the analyzed products at a confidence level of α = 0.05; the abbreviations used in the graph are described in Table 3).

The levels of antiradical activity of milk masses prepared from cocoa liquor from Ghana also maintained very similar relationships as in the case of the previous variant and the results obtained during the determination of polyphenols content (Figure 6). Again, the mixture with the addition of milk preparation Ml_5 (protein content 13.19%) was characterized by the highest value of the properties tested (in this case antiradical activity). A similar tendency of changes in antioxidant activity as in all previous determinations were shown on Figure 5 and Figure 6.

The antioxidant activity expressing the degree of reduction of free radicals by antioxidant compounds contained in the product is a valuable parameter of both the technological and health potential. The results obtained for all 21 milk masses allowed to determine in which of them the in vitro antioxidant potential was highest and how it changed depending on the raw materials used and the process parameters.

In the studies of Da Silva Medeiros, et al. [30], as well as Todorović, et al. [52], chocolate milk masses with declared cocoa liquor content of about 30% were distinguished by almost six times lower antioxidant activity potential, as compared to the tested bitter chocolates with declared cocoa liquor content of 67–70%. Similarly, the studies of Serafini, et al. [53] on blueberry fruit extracts and Dubeau, et al. [54] on different types of tea proved to confirm the dependence of lowering the antioxidant activity of extracts prepared after mixing them with milk. However, these values, unlike in the case of chocolates, were characterized by only 5–20% lower results compared to the activity of extracts not enriched with milk. Differences may result mainly from different protein content in milk added to tea (liquid—about 5 g/100 mL) and content of this ingredient in dried milk (about 30 g/100 g), as well as the amount of milk added in the mentioned variants.

In the investigated milk masses similar relationships were noted, because after the addition of milk or milk preparations, they reached much lower inactivation capacity of free radicals DPPH. This characteristic tendency is caused mainly by the fact, as indicated by Vertuani, et al. [55], that these masses are mixtures of various components, of which the cocoa liquor, bringing the greatest antioxidant character, is only a part of their composition. It is noteworthy, however, that this decrease was not as large as in the case of studies comparing milk chocolates to bitter chocolates. The differences were closer to those recorded for blueberry or tea extracts. This may mean that further processing and more intense industrial conditions, which were not provided for in this study, may lead to an increase in the reduction of antioxidant activity in milk chocolate. Intensified and extended by the process of tempering, the mixing of milk chocolate components causes more frequent interactions in the mixture of polyphenols with proteins, which, as indicated by Belščak, et al. [56], form complexes no longer having the same antiradical properties under the conditions of the antioxidant activity determination reaction. Similar conclusions were also drawn by Dubeau, et al. [54], who determined that casein proteins and catechin molecules in tea are mainly responsible for complex formation.

By the use of ANOVA analysis the results of antioxidant activity obtained for particular chocolate milk masses were compared to their polyphenols content. The correlation was close to 96.5%, which is a very strong correlation. It shows a simultaneous proportional decrease in antioxidant activity with a decrease in polyphenols content and its high value is also confirmed by Miller, et al. [57] in which this correlation reached almost 98%. However, more interesting from the nutritional point of view was a strong correlation between the results of protein content in masses and the content of previously discussed polyphenols or antioxidant activity. Negative correlation at the level of 0.66 and 0.76, respectively, showed a gradual proportional decrease in polyphenols and antioxidant activity with increasing protein content in the mass. The possible formation of protein–polyphenol complexes, supported by the study results and quoted in the literature, may explain this relationship. It can also be a valuable indication for producers of milk chocolate, who, while making chocolate products, also want to ensure pro-healthy values by preserving as many polyphenol compounds as possible. This relationship may also explain the observations of Serafini, et al. [53], who stated in their research that the reduction of antioxidant potential may also be influenced by the fat content, because taking into account whole, semiskimmed and skimmed milk, they obtained for blueberry fruit extracts a similarly characteristic decrease in antioxidant activity with increasing fat content. However, this dependence may have been largely due to the decrease in potential due to the antioxidant products of fat oxidation as well as to the decreasing antioxidant potential due to their combination with milk proteins.

Statistical inference was carried out with a 95% confidence level, to which the results of antioxidant activity of all tested milk chocolate masses were subjected. Fourteen homogeneous groups were obtained—one group more than for the analysis of polyphenols content (Table 5). Noteworthy is the analysis of masses T-7, T-8, T-1, T-12, T-16, and T-21, which were in the same groups in both the content of polyphenols and DPPH. A similar inference arises, as described in the analysis of polyphenol content. The analytical analysis confirmed the large variation between the results obtained, despite the fact that the tested masses can be grouped into three areas in terms of the used milk chocolate mass and in seven groups in terms of the used milk powder. The results indicate the complexity of aspects related to antioxidant potential.

Regardless of the decreasing antioxidant reduction potential in vitro studies, in many works, among others, Serafini, et al. [53], Loffredo, et al. [58], and Di Mattia, et al. [1], the same trend is not observed in studies on living organisms. In all these studies, the results of the increase in the occurrence of specific antioxidants in plasma consumed in the vicinity of milk did not differ significantly from those of milk-free counterparts. Such dependence may mainly result from the conditions of the digestive process of living organisms and many transformations and changes of substances that may occur as a result. Decreases in the amount determined in the body of epicatechin patients were recorded in studies by Neilson, et al. [59] after consumption of cocoa products containing milk. In each case, however, these values, although lower, did not show statistically significant differences. This phenomenon was explained by the variability of intestinal capture of food components and their subsequent transport between cells in patients’ organisms. It follows that lowering the antioxidant character of cocoa in a product with the addition of milk, such as milk chocolate, may be of primarily technological importance.

## 5. Conclusions

In this study, the highest content of polyphenols was determined in chocolate milk masses obtained from milk with the lowest protein content. This relationship is least evident in the masses obtained from cocoa liquor from Ghana, which also contained the least protein. Negative correlation between protein and polyphenol content in chocolate masses was observed.

A strong correlation was also shown for the decreasing ability of chocolate milk masses to scavenge DPPH radicals with increasing protein content of milk or milk preparations used to produce them. This correlation proves that the antioxidant potential of ready-made chocolate milk masses decreases with increasing content of milk proteins and consequently their ability to neutralize and inhibit the oxidation reaction of fats contained in chocolates decreases.

A strong correlation between the content of polyphenols in chocolate milk masses prepared for testing and their antioxidant activity was shown, which proves a proportional preservation of both values in the subject of the study.

In order to confirm this hypothesis, further studies should focus on the determination of fat oxidation products in masses and in vitro tests on the analyzed analytical matrix.

## Figures and Tables

**Table 1:** Dry matter and protein content in milk powder analyzed.

Milk Sample Number	Characteristics of Milk Type of Milk/Supplier/Milking Time/Production Technique	Protein Content (%)	Dry Matter Content (%)
1	WMP- MX/I 2018/D	28.53 ± 0.9	97.02 ± 0.08
2	WMP- MX/XII 2018/D	31.10 ± 1.1	97.35 ± 0.1
3	WMP- MX/IV 2017/D	30.35 ± 0.6	97.15 ± 0.2
4	WMP- MX/II 2018/D	27.44 ± 0.6	96.51 ± 0.1
5	WMP- MX/IX 2018/D	29.14 ± 0.8	96.72 ± 0.2
6	WMP- MX/VIII 2018/D	28.41 ± 1.0	96.34 ± 0.1
7	WMP- MX/V 2018/D	29.06 ± 0.8	97.31 ± 0.3
8	WMP- MX/IX 2018/D	29.00 ± 0.7	96.85 ± 0.09
9	WMP- MX/II 2018/D	27.99 ± 0.8	97.25 ± 0.1
10	WMP- MX/V 2018/D	28.86 ± 0.9	96.41 ± 0.2
11	WP- MX/III 2018/D	13.51 ± 1.1	97.91 ± 0.1
12	WMP- MX/I 2018/D	29.47 ± 0.9	95.92 ± 0.3
13	MP - MX/IV 2018/C (consisting in 80% of WM, enriched with lactose and WP)	18.90 ± 0.8	96.55 ± 0.09
14	MP - MX/IV 2018/C (consisting of 47% sugar and the remainder of milk, permeate and cream)	13.19 ± 1.0	97.26 ± 0.1
15	WMP- MX/VII 2018/D	25.78 ± 1.1	95.32 ± 0.2
16	WMP- MX/VI 2018/D	26.43 ± 0.9	95.17 ± 0.2
17	WMP- MX/V 2018/D	27.02 ± 0.6	96.24 ± 0.3
18	WMP- MZ/V 2018/D	26.76 ± 0.7	97.56 ± 0.09
19	WP- MZ/V 2018/D	11.61 ± 0.9	97.14 ± 0.1

The results of the protein content and dry weight given in grams in 100 g of product (g 100 g^−1^) were expressed in (%)—(*w/w*). Whole-milk powder—WMP, Whey powder—WP, Milk preparation—MP, manufacturer X—MX, manufacturer Y—MY, manufacturer Z—MZ, drying technique—D, cylindrical drying technique—C.

**Table 2:** List and marking of raw materials used to prepare chocolate milk masses.

Sample Code	Characteristics of the Test Material
**Ch_1**	Cocoa liquor/unroasted beans/Peru,
**Ch_2**	Cocoa liquor/roasted beans/Ivory Coast
**Ch_3**	Cocoa liquor/roasted beans/Ghana
**Ml_1**	WMP- MX/IV 2017/D
**Ml_2**	WMP/- MX/II 2018/D
**Ml_3**	WMP/- MX/IX 2018/D
**Ml_4**	MP – MX/IV 2018/C (consisting in 80% of WM, enriched with lactose and WP)
**Ml_5**	MP – MX/IV 2018/C (consisting of 47% sugar and the remainder of milk, permeate and cream)
**Ml_6**	WMP- MX/VII 2018/D
**Ml_7**	WMP- MZ/V 2018/D

Whole-milk powder—WMP, Whey powder—WP, Milk preparation—MP, manufacturer X—MX, manufacturer Y—MY, manufacturer Z—MZ, drying technique—D, cylindrical drying technique—C.

**Table 3:** List and marking of chocolate milk masses.

Sample Code	Characteristics of Chocolate Milk Masses	Sample Code	Characteristics of Chocolate Milk Masses
**T-1**	CMM prepared using Ch_1 and Ml_1	**T-12**	CMM prepared using Ch_2 and Ml_5
**T-2**	CMM prepared using Ch_1 and Ml_2	**T-13**	CMM prepared using Ch_2 and Ml_6
**T-3**	CMM prepared using Ch_1 and Ml_3	**T-14**	CMM prepared using Ch_2 and Ml_7
**T-4**	CMM prepared using Ch_1 and Ml_4	**T-15**	CMM prepared using Ch_3 and Ml_1
**T-5**	CMM prepared using Ch_1 and Ml_5	**T-16**	CMM prepared using Ch_3 and Ml_2
**T-6**	CMM prepared using Ch_1 and Ml_6	**T-17**	CMM prepared using Ch_3 and Ml_3
**T-7**	CMM prepared using Ch_1 and Ml_7	**T-18**	CMM prepared using Ch_3 and Ml_4
**T-8**	CMM prepared using Ch_2 and Ml_1	**T-19**	CMM prepared using Ch_3 and Ml_5
**T-9**	CMM prepared using Ch_2 and Ml_2	**T-20**	CMM prepared using Ch_3 and Ml_6
**T-10**	CMM prepared using Ch_2 and Ml_3	**T-21**	CMM prepared using Ch_3 and Ml_7
**T-11**	CMM prepared using Ch_2 and Ml_4		

Chocolate milk masses—CMM, other code explanation in Table 2.

**Table 4:** Cocoa liquor—content: dry matter, protein, polyphenols, and antioxidant activity (code explanation in Table 2).

Sample Code	Dry Matter	Proteins	Polyphenols	Antioxidant Activity
	% (*w/w*)	% (*w/w*)	mg GAE 100 g^−1^	% (*w/w*)
**Ch_1**	97.75 ± 0.3	13.62 ± 1.2	3284.4 ± 20.3	93.5 ± 2.4
**Ch_2**	98.92 ± 0.6	14.67 ± 1.7	2881.3 ± 29.5	91.6 ± 3.6
**Ch_3**	98.96 ± 0.4	14.25 ± 1.6	2723.6 ± 18.6	90.2 ± 4.3

**Table 5:** Results of statistical analysis of polyphenols content and antiradical activity of all analyzed milk chocolate masses (code explanation in Table 3).

Codes of Masses	Polyph.	DPPH	Codes of Masses	Polyph.	DPPH	Codes of Masses	Polyph.	DPPH
**T-1**	J	h	**T-8**	A	a	**T-15**	B C	b
**T-2**	K	j	**T-9**	C G	c	**T-16**	D E	de
**T-3**	J	ij	**T-10**	A B	b	**T-17**	C D	c
**T-4**	L	m	**T-11**	G H	gh	**T-18**	G	ij
**T-5**	M	n	**T-12**	I	i	**T-19**	H	k
**T-6**	K	l	**T-13**	E F	de	**T-20**	F	fg
**T-7**	K	k	**T-14**	D E	cd	**T-21**	E F	ef

The same letter A, B or a, b means that there are no statistically significant differences between the analyzed products at a confidence level of α = 0.05; the abbreviations used in the graph are described in Table 3.
